# Retrorectal Hernia: A Rare Cause of Constipation Diagnosed on Magnetic Resonance (MR) Defecography

**DOI:** 10.7759/cureus.58848

**Published:** 2024-04-23

**Authors:** Olanrewaju Ogunleye, Abeer H Abdelhafez, Eduardo Matta, Larry Kramer

**Affiliations:** 1 Department of Radiology, University of Texas Health Science Center at Houston, Houston, USA

**Keywords:** rectocele, mr defecography, sigmoid colon, rectum, hernia

## Abstract

Retrorectal herniation of the sigmoid colon is a rare condition characterized by the protrusion of a segment of the colon into the pre-sacral space and posterior to the rectum. This herniation occurs through a defect in the peritoneum, which may have developed secondary to congenital mechanisms, surgery, trauma, or inflammatory processes. Here, a case of retrorectal herniation of the sigmoid colon in an elderly female patient presenting with constipation is reported, with a review of the literature.

## Introduction

Retrorectal hernias are a rare type of internal hernia, with only a few cases reported in the literature [1,2]. Retrorectal herniation of the sigmoid colon is the protrusion of a segment of the sigmoid colon through a defect in the peritoneum into the pre-sacral space and posterior to the rectum [1,3]. This peritoneal defect can be secondary to congenital mechanisms, surgery, trauma, or inflammatory processes [4]. As with other internal hernias, patients may develop nonspecific chronic symptoms such as abdominal pain, constipation, and obstipation. More severe symptoms may include acute bowel obstruction, intermittent bowel obstruction, peritonitis, and sepsis [4,5]. We present a case of a 73-year-old female patient with a finding of retrorectal hernia of the sigmoid colon.

## Case presentation

**Figure 1:**
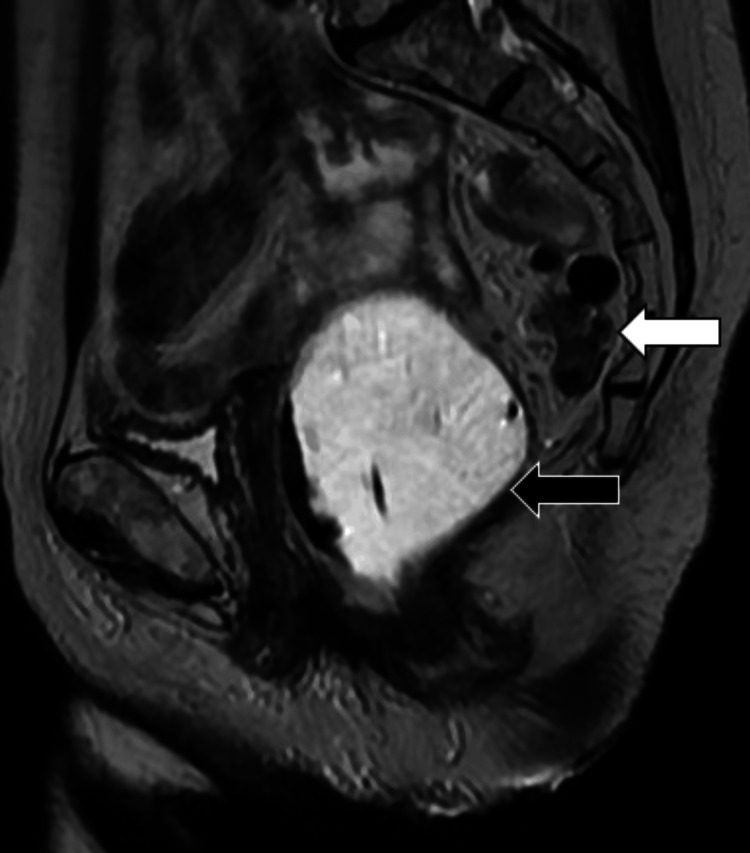
Sagittal T2-weighted image of the pelvis showing the sigmoid colon (white arrow) in the presacral space – posterior to the rectum (black arrow) during the rest phase of MR defecography. The rectum is distended with T2 hyperintense gel per MR defecography protocol.

**Figure 2:**
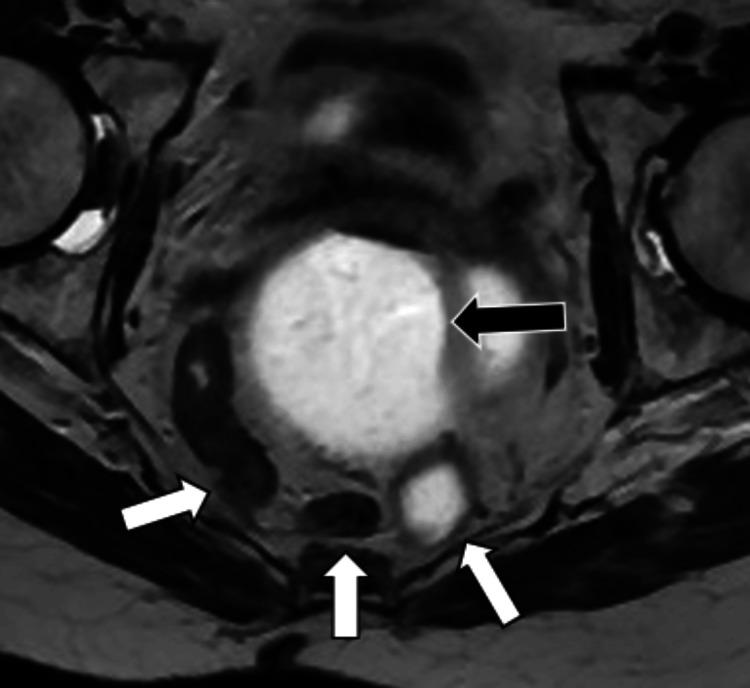
Axial T2-weighted image of the pelvis during MR defecography showing loops of the sigmoid colon (white arrows) in the presacral space – posterior to the distended rectum (black arrow).

**Figure 3:**
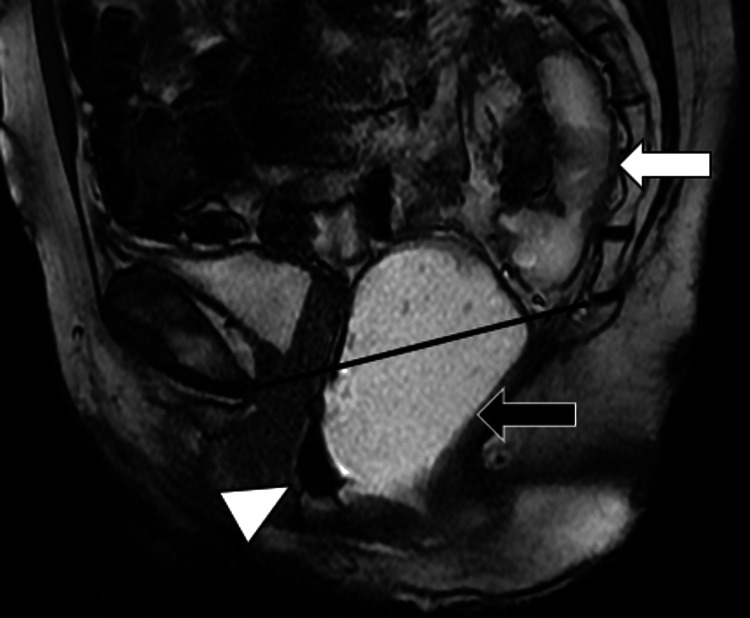
Sagittal T2-weighted image of the pelvis showing persistent retrorectal herniation of the sigmoid colon (white arrow) and pelvic floor laxity during the evacuation phase of MR defecography. The distended rectum (black arrow) descends below the pubococcygeal line (black line). An anterior wall rectocele is seen (white arrowhead).

A 73-year-old female patient, with a history of diverticulosis and prior hysterectomy, was referred to our department for magnetic resonance defecography following complaints of constipation. MR imaging revealed herniation of the sigmoid colon within the presacral space, posterior to the rectum (Figures 1, 2). There was no evidence of bowel obstruction. A moderate pelvic floor laxity with moderate anorectal descent and a 2 cm anterior wall rectocele were also reported during the straining and evacuation phases (Figure 3). The patient was subsequently seen by the referring physician and managed conservatively.

## Discussion

Retrorectal hernia is extremely uncommon, with limited reports of cases encountered in published scientific literature [1,6]. Retrorectal herniation is the pelvic floor protrusion of bowel loops through a defect in the peritoneum into the retrorectal space or other extraperitoneal spaces such as the Douglas’ and Retzius’ spaces [6]. Retrorectal herniation of the sigmoid colon is the protrusion of a segment of the sigmoid colon into the presacral space and posterior to the rectum through a defect in the peritoneum [1].

The symptoms of herniation vary from mild, non-specific chronic complaints such as vague abdominal pain, abdominal distension, pelvic pain, and constipation to more severe symptoms such as intermittent colicky pain and recurrent intestinal obstruction [5,7]. The severity of symptoms experienced by subjects also varies, mostly depending on the duration of herniation, the hernia’s reducibility, and the development of complications. Patients with untreated large bowel obstruction may develop severe dehydration, peritonitis, or sepsis [8,9].

Like other internal hernias, retrorectal hernias tend to reduce spontaneously and are best imaged when symptomatic [10]. Retrorectal hernias, like other pelvic hernias, can be assessed by various imaging modalities, including fluoroscopy, ultrasound, and MR defecography. Fluoroscopy has long been regarded as the gold standard, but major limitations include its invasive nature and the use of potentially harmful ionizing radiation [11].

MR defecography is a safe, relatively non-invasive imaging method that enables the acquisition of multiplanar and multiparametric images, which assist physicians in evaluating pelvic compartments without exposure to potentially dangerous ionizing radiation [12]. Compared to other modalities, MR defecography is superior in evaluating the preoperative static and dynamic pelvic anatomy of patients with pelvic pathologies [13].

## Conclusions

In conclusion, this case report details the finding of a rare case of retrorectal hernia in a patient with a complaint of constipation. The diagnosis of retrorectal hernia was made during pre-treatment MR defecography. The management of retrorectal herniation depends on the severity of the patient’s symptoms and varies from conservative to surgical reduction.
